# Assessment of hard tissue changes after horizontal guided bone regeneration with the aid of deep learning CBCT segmentation

**DOI:** 10.1007/s00784-024-06136-w

**Published:** 2025-01-13

**Authors:** Daniel Palkovics, Alexandra Hegyi, Balint Molnar, Mark Frater, Csaba Pinter, David García-Mato, Andres Diaz-Pinto, Peter Windisch

**Affiliations:** 1 Department of Periodontology, Semmelweis University, Budapest, Hungary; 2 Dent.AI Medical Imaging Ltd, Budapest, Hungary; 3 Department of Operative and Esthetic Dentistry, Faculty of Dentistry, University of Szeged, Szeged, Hungary; 4 Empresa de Base Technológica Internacional de Canarias, S.L. (EBATINCA), Las Palmas De Gran Canaria, Spain; 5 School of Biomedical Engineering & Imaging Sciences, King’s College London, London, UK

**Keywords:** CBCT segmentation, Deep learning, Convolutional neural networks, 3D evaluation, Guided bone regeneration, Surgical planning

## Abstract

**Objectives:**

To investigate the performance of a deep learning (DL) model for segmenting cone-beam computed tomography (CBCT) scans taken before and after mandibular horizontal guided bone regeneration (GBR) to evaluate hard tissue changes.

**Materials and methods:**

The proposed SegResNet-based DL model was trained on 70 CBCT scans. It was tested on 10 pairs of pre- and post-operative CBCT scans of patients who underwent mandibular horizontal GBR. DL segmentations were compared to semi-automated (SA) segmentations of the same scans. Augmented hard tissue segmentation performance was evaluated by spatially aligning pre- and post-operative CBCT scans and subtracting preoperative segmentations obtained by DL and SA segmentations from the respective postoperative segmentations. The performance of DL compared to SA segmentation was evaluated based on the Dice similarity coefficient (DSC), intersection over the union (IoU), Hausdorff distance (HD95), and volume comparison.

**Results:**

The mean DSC and IoU between DL and SA segmentations were 0.96 ± 0.01 and 0.92 ± 0.02 in both pre- and post-operative CBCT scans. While HD95 values between DL and SA segmentations were 0.62 mm ± 0.16 mm and 0.77 mm ± 0.31 mm for pre- and post-operative CBCTs respectively. The DSC, IoU and HD95 averaged 0.85 ± 0.08; 0.78 ± 0.07 and 0.91 ± 0.92 mm for augmented hard tissue models respectively. Volumes mandible- and augmented hard tissue segmentations did not differ significantly between the DL and SA methods.

**Conclusions:**

The SegResNet-based DL model accurately segmented CBCT scans acquired before and after mandibular horizontal GBR. However, the training database must be further increased to increase the model’s robustness.

**Clinical relevance:**

Automated DL segmentation could aid treatment planning for GBR and subsequent implant placement procedures and in evaluating hard tissue changes.

## Introduction

Advances in digital technology have significantly impacted medicine, continuously shaping the course of interventions and clinical decision-making. In dentistry and oral surgery, this technological progress has enabled more efficient diagnosis and personalized treatment approaches. Various forms of computer technologies, such as digital imaging, 3D modeling, additive manufacturing, and artificial intelligence (AI), contribute to more predictable dental surgical procedures, reducing the occurrence of intra- and post-operative complications.

AI is a term that collectively refers to machines, programs, and algorithms designed to perform tasks typically performed by humans that can mimic human thinking [1]. The most relevant AI tools in healthcare are machine learning, natural language processing, expert systems, speech recognition, and robotics [2]. AI technologies have developed significantly since the second AI winter [3], resulting in their integration into routine medical care. AI technologies are most frequently used for clinical decision-making, prediction, image analysis, and robot-assisted procedures [2, 4–6]. In dentistry, the most common AI applications are radiographic image analysis and processing (segmentation).

The segmentation of volumetric imaging modalities, such as computed tomography or cone-beam computed tomography (CBCT), aims to reconstruct relevant anatomical structures in three dimensions (3D) from planar image datasets [7]. In oral surgery [8], maxillo-facial surgery [9], periodontology [10], and implantology [11], preoperative data processing enables the 3D virtual planning of surgical interventions. Radiographic images are mainly segmented with either global thresholding [12] or various semi-automatic (SA) methods [13]. The global thresholding method is fast but inaccurate since it cannot differentiate individual anatomical structures. Therefore, 3D models prepared with this method are unsuitable for surgical planning. In contrast, SA segmentation methods can produce highly detailed 3D models; however, they are very time-consuming and have a relatively steep learning curve. With the development of AI, automatic deep learning (DL) segmentation is becoming increasingly accessible.

Over the past five years, several articles have been published on the automatic segmentation of dental CBCT images. Developing an AI-based segmentation model for dental CBCT scans is challenging due to the wide variation in patients’ anatomy, number of remaining teeth, the diversity of hard tissue defects, and the frequent presence of metal artifacts. In order to ensure the robustness of the AI model, previous segmentation methods have used CBCT scans from fully dentate patients for training to increase homogeneity [14, 15] or have included an excessive amount of CBCT scans in the training database [16]. For the automatic segmentation of CBCT images, the OMFS-IMPATH research group (Leuven, Belgium; https://omfsimpath.be/) has used a convolutional neural network (CNN) with a 3D U-Net architecture [14, 15]. Commonly used for 3D radiographic image analysis, the 3D U-Net architecture features a two-arm encoder-decoder structure similar to a standard U-Net. Compared to SA segmentation, the developed DL segmentation model achieved an intersection over union (IoU) value of 0.94 for the segmentation of the mandible on dental CBCT scans [15]. On the other hand, Cui et al. [16, 17] developed a multi-stage volumetric U-Net (V-Net) model trained on a database consisting of almost 5000 CBCT scans derived from routine clinical practice. Their model achieved 91.5% accuracy for tooth segmentation and 93.0% for alveolar bone segmentation [16].

Our group has previously developed a multi-stage DL model for segmenting dental CBCT scans [18] using the PyTorch-based open-source MONAI framework with a network architecture based on SegResNet, as proposed by Myronenko et al. [19]. Compared to an SA segmentation method, this model achieved an IoU value of 0.91 in the segmentation of partially edentulous mandibles on CBCT scans [20]. The acquired 3D models could be later used for digital surgical planning of guided bone regeneration (GBR) procedures [21]. However, the performance of the developed SegResNet-based DL segmentation model has not been tested on post-GBR CBCT scans. Automatic segmentation of the augmented area might be challenging due to differences in the radiographic image of augmented bone and local artifacts produced by titanium pins.

The CBCT segmentation and 3D model acquisition include the ability to perform dental implant placement following GBR using a realistic virtual patient model. Accurate segmentation of the augmented area can facilitate more accurate treatment planning and the 3D positioning of dental implants. Another advantage of accurate post-GBR CBCT scan segmentation is the ability to assess surgical outcomes in 3D. Our previous articles have described a methodology that allows for detailed volumetric and 3D morphological assessments of surgical outcomes [22, 23]. The 3D models of CBCT scans allow a deeper understanding of tissue alterations that occur after reconstructive procedures. However, the lengthy SA segmentation process presents a significant barrier to its routine use by clinicians and researchers. Applying a DL-based segmentation model could reduce the time required for the evaluation process, enabling routine 3D volumetric evaluation of surgical procedures.

Therefore, this study aimed to evaluate the performance of a SegResNet-based DL model in segmenting CBCT scans acquired before and six months after horizontal GBR procedures. Its secondary aim was to assess the SegResNet-based DL model’s accuracy in determining the volumetric hard tissue gain after horizontal GBR procedures.

## Methods

### Study design

This study consisted of three main phases: (i) building the training database, (ii) training and validating the SegResNet-based DL model, and (iii) testing its accuracy. The SegResNet-based DL model was developed using 70 CBCT scans (57 for training, 13 for validation). The CBCT scans were derived from routine clinical practice of partially edentulous patients undergoing periodontal rehabilitation and were seeking for implant-borne prosthetic solutions. During testing, the segmentation results of the SegResNet-based DL model were compared to the results of an SA method, which was considered the ground truth. SA segmentations were performed by a calibrated rater who participated in a calibration session prior to the beginning of the study. Testing was performed on 10–10 CBCT scans acquired before and after mandibular horizontal GBR procedures. The proposed multi-stage SegResNet-based DL model segmented and classified teeth; however, the current paper only reports the accuracy of its mandible segmentation.

This study was conducted according to the Declaration of Helsinki, as revised in 2013 [24], and its protocol was approved by the Regional Institutional Scientific and Research Ethics Committee of Semmelweis University (approval number: SE RKEB 138/2023). The examination was conducted according to the guidelines proposed by Schwendicke et al. [25]. All CBCT data used for training, validation, and testing were anonymized.

### Preparation of the training database

The training database was compiled from the CBCT scans of patients treated at the Department of Periodontology of Semmelweis University. The CBCT scans of patients with dental implants and fully edentulous patients were excluded from the training database. However, the CBCT scans of patients with restorations, root canal-treated teeth, or titanium pins from previous reconstructive surgeries were included in the training database. Only CBCT images containing the complete mandibular dental arch were included.

The CBCT scans included in the training database were acquired with the following parameters:

- I-CAT FLX^®^ (KaVo Dental GmbH, Biberach an de Riß, Germany): voxel size = 300 μm, voltage = 120 kV, tube current = 36 mA, scan time: 8.9 s;
- Planmeca Viso^®^ (Planmeca, Helsinki, Finland): voxel size = 150 μm, voltage = 100 kV, tube current = 12 mA, scan times: 5.04–8 s.

The selected CBCT scans were imported into 3D Slicer [26], an open-source image-processing software platform for segmentation. All training datasets were reformatted to a slice interval of 0.2 mm x 0.2 mm x 0.2 mm. The alveolar bone was segmented using a region-growing algorithm (Growing from seeds). Teeth were segmented using the watershed method. Errors were corrected using manual tools (Paint, Erase, and Scissors). The infraalveolar nerve was segmented using the “Draw tube” effect. After segmentation, the labels were renamed using a uniform terminology and color coding.

### SegResNet DL architecture

**Fig. 1:**
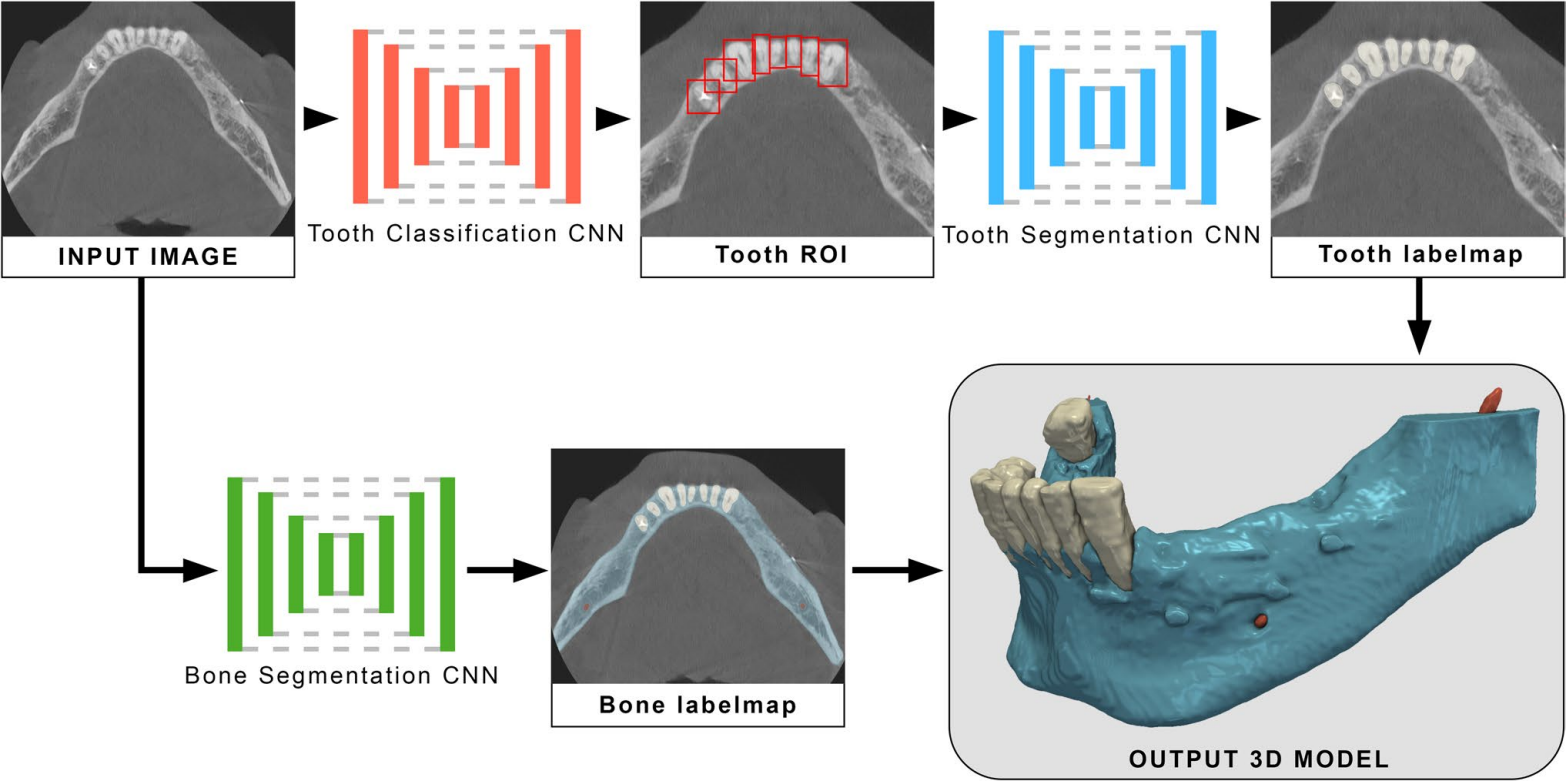
Architecture of the developed multi-stage SegResNet-based deep learning (DL) model

The developed model used a multi-stage DL framework to automatically segment CBCT images. It consisted of two stages: the first stage, segmentation and classification of the teeth was performed, and the second performed the segmentation of the alveolar bone and inferior alveolar nerves. The multi-stage SegResNet-based DL model was trained using MONAI (https://github.com/Project-MONAI/MONAI). Like a 3D U-Net, this network also has an encoder-decoder-based CNN architecture with an asymmetrically larger encoder to extract image features and a smaller decoder to reconstruct the segmentation mask of the anatomical structures of interest. The encoder uses ResNet [27] blocks, each consisting of two convolutions with normalization and rectified linear units (ReLU). It follows a common CNN approach to progressively down-size image dimensions. The number of down-sampling blocks in each layer was 1, 2, 2, 2, and 4, respectively. All convolutions are 3 × 3 × 3 with 32 initial filters. The decoder is structured like the encoder but with a single block per spatial level. Each decoder level begins with upsizing (reducing the number of features by a factor of 2 and doubling the spatial dimension), followed by adding an encoder output of the equivalent spatial level. The output image at the end of the decoder has the same spatial size as the original image, and the number of features is equal to the initial input feature size, followed by 1 × 1 × 1 convolution into three channels and a sigmoid function. For training 57 CBCT scans, for validation 13 CBCT scans were used (Fig. 1).

### Testing

During testing, DL segmentations were compared to the ground truth SA segmentations. The sample size was determined based on Tao et al. [28] using dependent sample *t*-tests, considering an α of 0.05, power (1 − β) of 0.80, and effect size of 1.027. Based on the power calculations, the performance of the SegResNet-based DL model was tested on 10–10 CBCTs acquired before and after mandibular horizontal GBR. The sample size calculations were performed using the G*Power 3.1.9.7 software [29, 30].

Four parameters were used to assess the differences between DL and SA segmentation:

- True positive (TP): number of correctly segmented voxels;
- True negative (TN): number of correctly NOT segmented voxels;
- False positive (FP): number of incorrectly segmented voxels;
- False negative (FN): number of missed voxels.

Ground truth segmentations were acquired using the same SA segmentation method described in the “Preparation of the training database” section.

### Consistency

To assess intra-rater reliability (IRR), a single rater participated in two calibration sessions 14 days apart to determine the consistency of repeated measurements. The calibrated rater performed segmentation of mandibles on CBCT scans (independent from the study sample) from five patients taken before and after GBR. Since data were found to be normally distributed, Pearson’s correlation coefficient (PCC) was calculated to assess the IRR. Volumes of segmented mandibles were compared for IRR assessment.

### Timing

The duration of DL and SA segmentations, measured in seconds, was compared. For DL segmentation, the timeframes for tooth and mandible segmentation were recorded separately; however, only the mandible segmentation duration was included in the comparison with SA segmentation.

### GBR procedure

CBCT scans in the testing set were obtained from patients who underwent horizontal GBR procedures. Alveolar ridge defects were treated using a split-thickness flap design according to Windisch et al. [31]. The edentulous area was grafted using a 1:1 mixture of autogenous bone chips and bovine-derived xenografts (cerabone^®^; Botiss GmbH, Berlin, Germany). The grafted area was covered with a long-term resorbable pericardium membrane (Jason^®^; Botiss GmbH, Berlin, Germany) which was fixed using titanium membrane fixation screws (Membrane-Screw, 1.5 × 4 mm; Ustomed Instrumente GmbH, Tuttlingen, Germany).

### Assessing volumetric hard tissue changes using DL segmentation

**Fig. 2:**
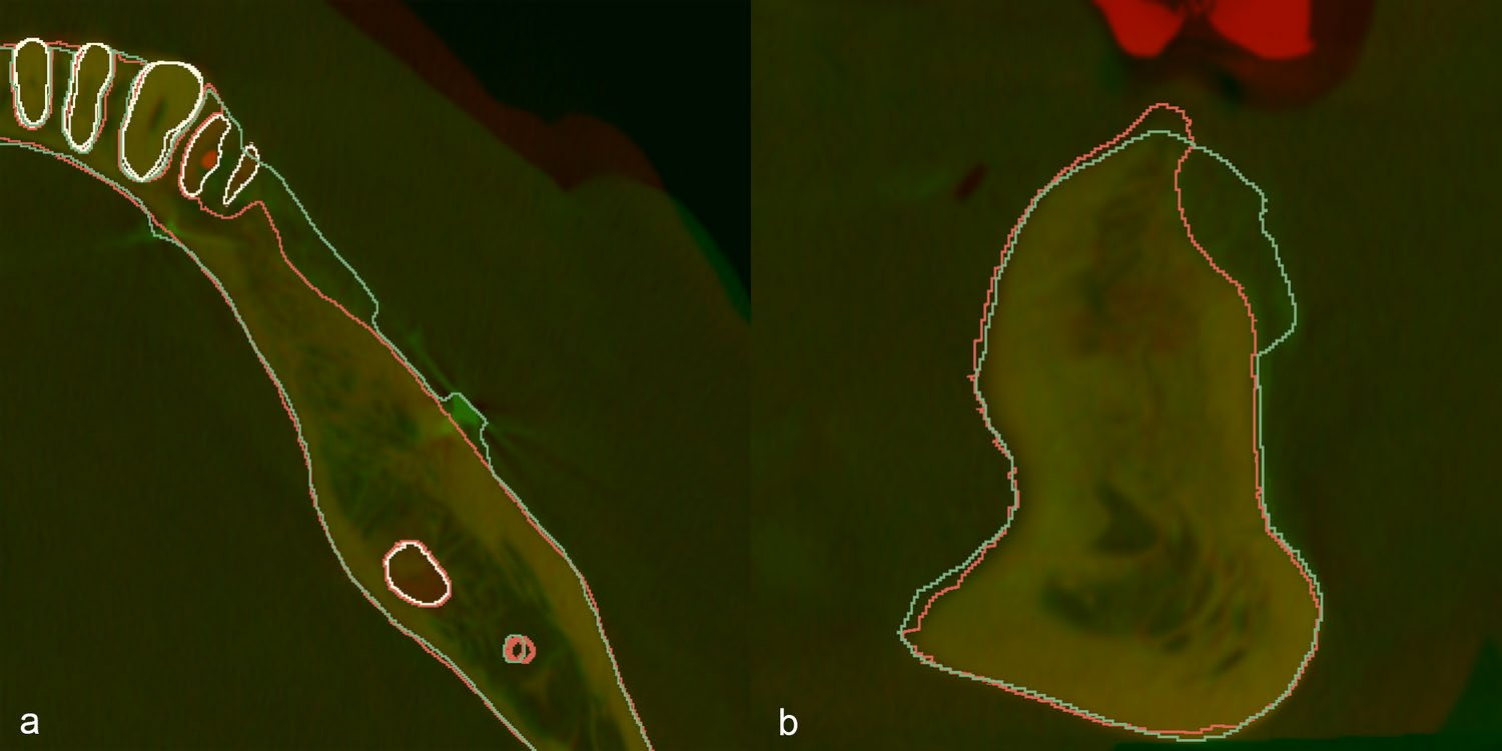
Alignment of preoperative and postoperative cone-beam computed tomography (CBCT) scans. The red labelmap shows the outline of preoperative CBCT scans, and the green labelmap shows the outline of postoperative CBCT scans A: Axial view B: Coronal view

After SA and DL segmentation, the pre- and post-operative CBCT scans were aligned using the “elastix” extension of 3D slicer [32] which is an automatic voxel-intensity-based registration algorithm. After spatial alignment of CBCT images preoperative DL and SA segmentations were subtracted from those of the postoperative DL and SA segmentations, respectively to obtain 3D models of the augmented hard tissues. Pre- and postoperative CBCT scans were subtracted using the “Logical operators” tool in the “Segment Editor” module of 3D Slicer [7]. By comparing the 3D models acquired by subtraction of DL and SA segmentations, the SegResNet-based DL model’s performance in segmenting augmented hard tissues could be assessed (Fig. 2).

### Outcome variables

Based on the four parameters, the primary outcome was the Dice similarity coefficient (DSC), which represents the proportion of overlapping voxels between DL and SA segmentations relative to their union. DSC values range from 0 (no overlap) to 1 (perfect overlap).

$$\:DSC=\frac{2\times\:TP}{2\times\:TP+FP+FN}$$

Like the DSC, the IoU also reflects the proportion of overlapping voxels between the two segmentations relative to the union of the voxels from both segmentations:

$$\:IoU=\frac{TP}{TP+FP+FN}$$

The 95th percentile Hausdorff distance (HD95) reflects the maximum of the minimum distances between the DL and SA segmentations at the 95th percentile; an HD95 of 0 mm reflects complete overlap. The volumes of the mandibular 3D models generated with DL- and SA segmentations and their differences were measured in cubic centimeters (cm^3^). All five outcome measures were determined for the baseline segmentations, six-month follow-up segmentations, and the newly-formed, augmented hard tissues.

### Statistical analysis

All variables are presented as the mean ± standard deviation. Statistical significance was assessed using inferential statistics, with a *p* < 0.05 considered statistically significant. The normality of the examined variables was assessed using the Shapiro–Wilk test. As the variables were found to be normally distributed, they were compared using parametric statistical tests. The volumes of the mandible models acquired with DL and SA segmentations and their volumetric differences were compared using Student’s paired samples *t*-test. Durations of DL and SA segmentations were also compared using a Student’s paired samples *t*-test. All statistical calculations were performed using the STATA software package (release 18; StataCorp LLC, College Station, TX, USA).

## Results

### Consistency

The Pearson’s correlation for IRR showed a strong positive correlation between the two sets of segmentation volumes, with a PCC of 0.97. This value indicates excellent consistency within the rater’s segmentations across measurements. The correlation is statistically highly significant (*p* < 0.0001), confirming that the rater’s repeated segmentations are highly reliable.

### Preoperative CBCT segmentation

**Fig. 3:**
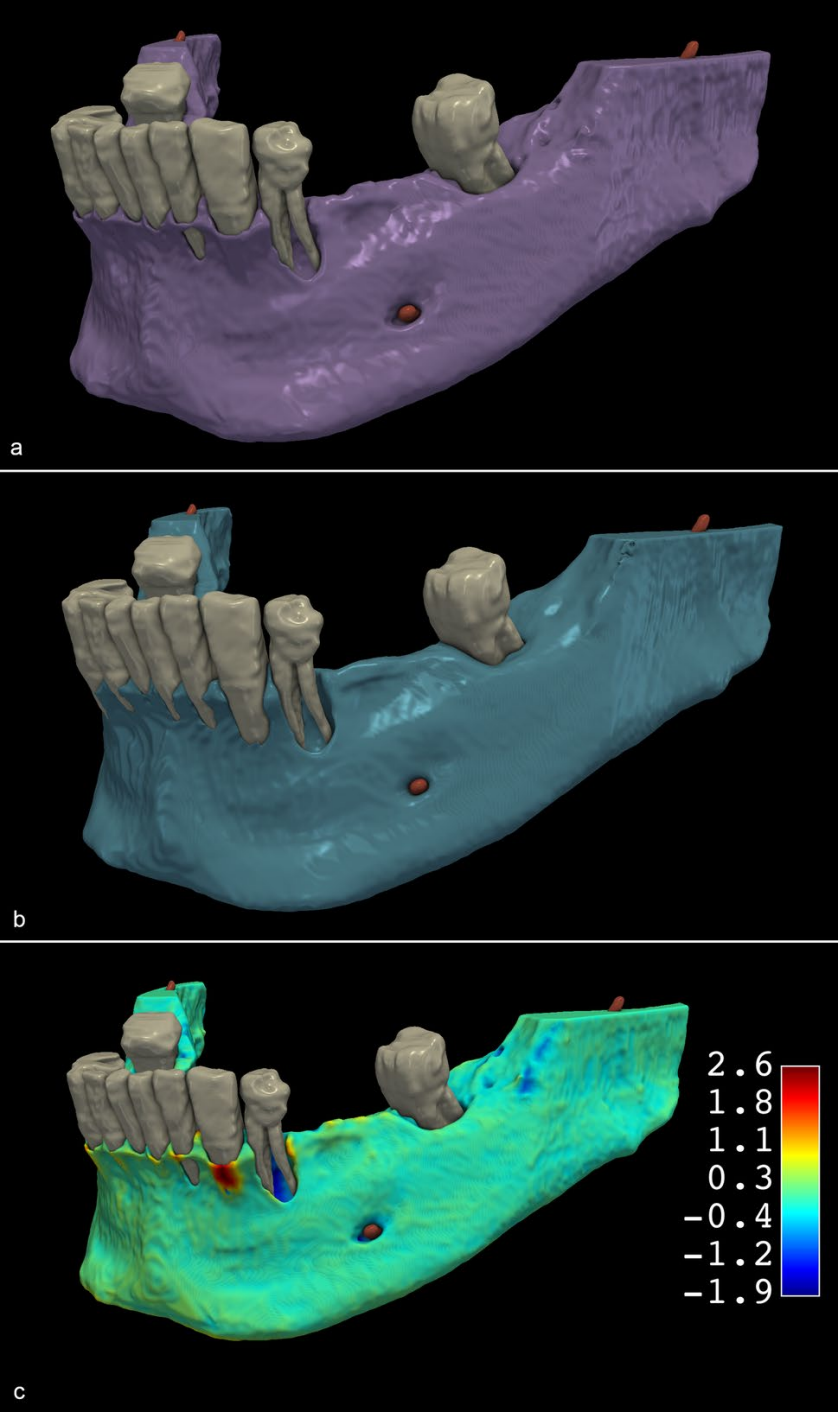
Comparison of preoperative mandible segmentations obtained by deep learning (DL) and semi-automatic (SA) segmentations A: SA segmentation results B: DL segmentation results C: Colormap model visualizing the differences between the DL and SA methods. Red areas represent positive differences, and blue areas represent negative differences between the reference SA and developed DL models

**Fig. 4:**
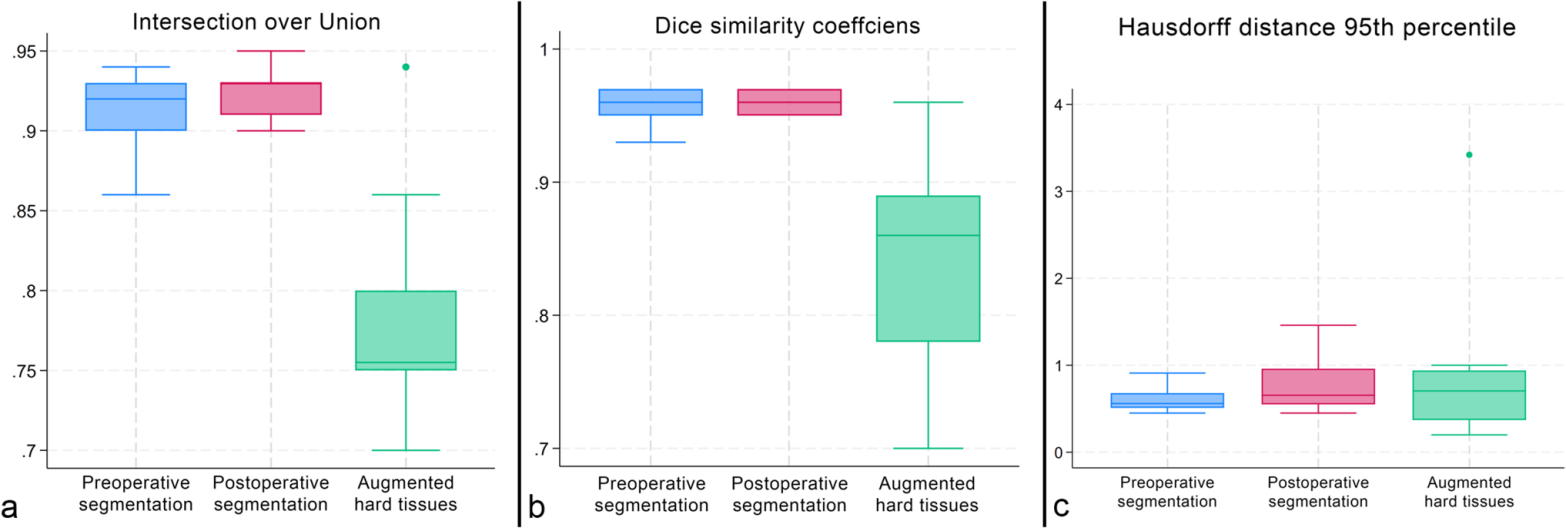
Box plots A: Distribution of intersection over union (IoU) values B: Distribution of Dice similarity coefficients (DSC) C: Distribution of 95th percentile Hausdorff distances (HD95)

**Fig. 5:**
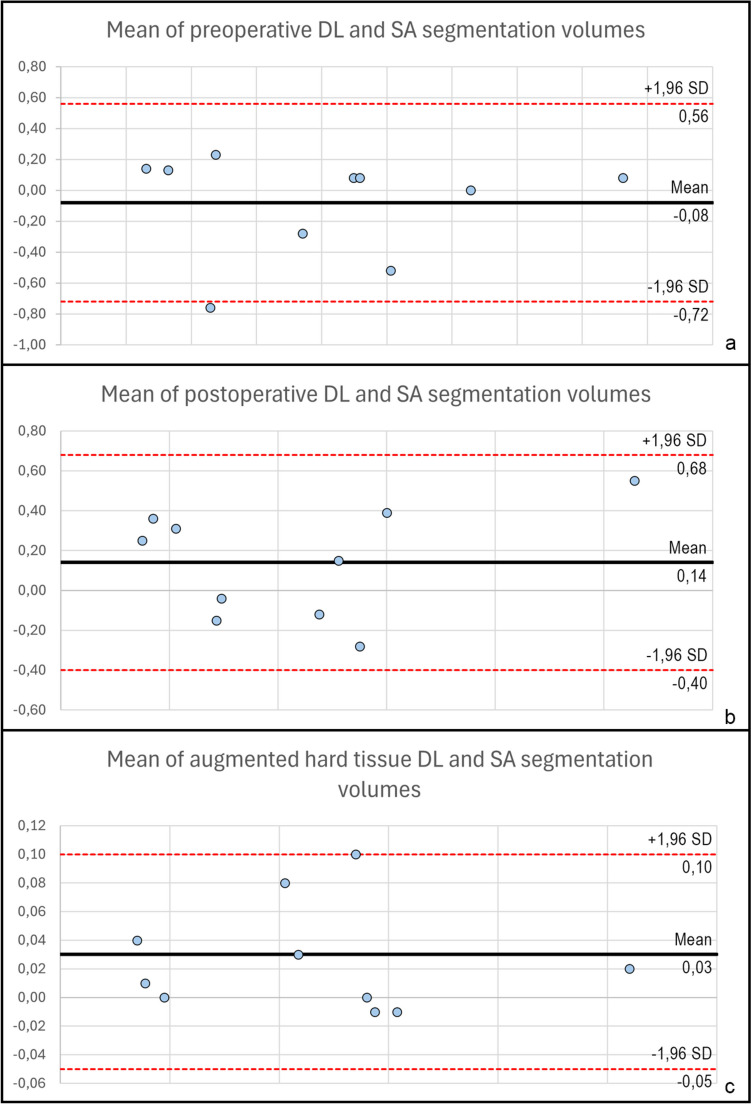
Bland-Altman plot of mean differences in segmentation volumes A: Mean volume differences of preoperative segmentations B: Mean volume differences of postoperative segmentations C: Mean volume differences of augmented hard tissue segmentations

The mean DSC between the DL and SA segmentations of the mandible on CBCT scans acquired before GBR was 0.96 ± 0.01 (Fig. 3). Like the DSC, the mean IoU, reflecting the spatial overlap between the DL and SA segmentations, was 0.92 ± 0.02. The mean HD95, reflecting the maximum of the minimum distances, was 0.62 mm ± 0.16 mm. The distributions of the IoU, DSC, and H95 data were visualized as box plots (Fig. 4A). Student’s paired samples *t*-test indicated no statistically significant difference in the total volume of DL and SA mandible segmentations (*p* = 0.45). The mean volume was 20.22 cm^3^ ± 11.39 cm^3^ for the DL segmentations and 20.14 cm^3^ ± 11.40 cm^3^ for the SA segmentations. The mean differences between the volumes of SA and DL segmentations were visualized in a Bland–Altman plot (Fig. 5A). The data are summarized in Table 1.

**Table 1:** Preoperative CBCT segmentation

	DSC^a^	IoU^b^	H95^c^ (mm)	DL volume^d^ (cm^3^)	SA volume^e^ (cm^3^)
Mean	0.96	0.92	0.62	20.22	20.14
St. dev.^f^	0.01	0.02	0.16	11.39	11.40
Minimum	0.93	0.83	0.45	6.45	6.59
Maximum	0.97	0.94	0.91	43.07	43.15
Median	0.96	0.92	0.56	20.55	20.45
*p*-value	na	na	na	0.45^g^	

^a^Dice similarity coefficient, ^b^Intersection over union, ^c^95th percentile Hausdorff distance, ^d^Volume of deep learning segmentation, ^e^Volume of semi-automatic segmentation, ^f^Standard deviation, ^g^Student’s paired samples *t*-test

### Postoperative CBCT segmentation

**Fig. 6:**
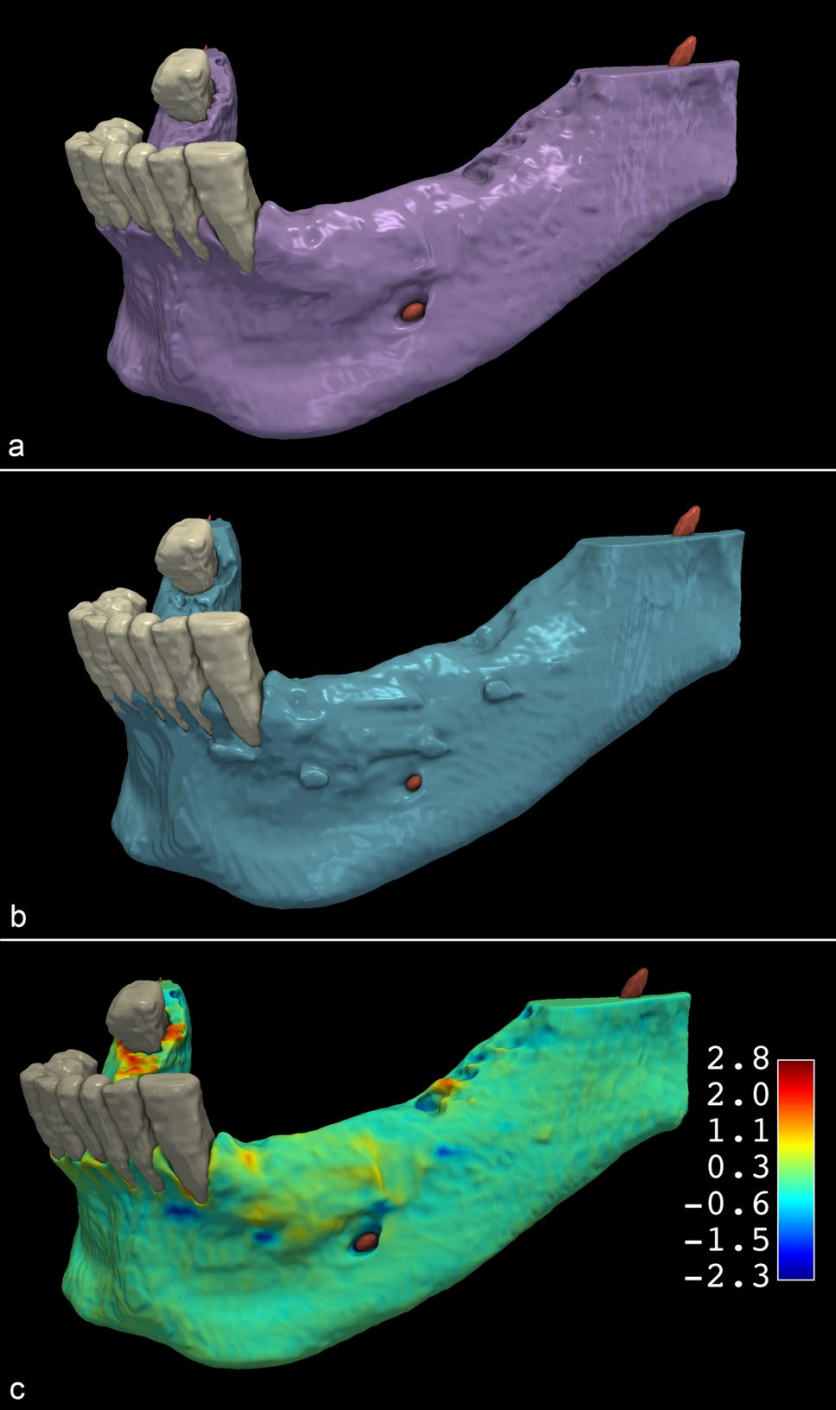
Comparison of postoperative mandible models obtained by DL and SA segmentations A: SA segmentation results B: DL segmentation results C: Colormap model visualizing the differences between the two methods. Red areas represent positive differences, and blue areas represent negative differences between the reference SA and developed DL models

Regarding the six-month follow-up CBCT scans, the mean DSC was 0.96 ± 0.01, and the mean IoU was 0.92 ± 0.02 between the DL and SA segmentations (Fig. 6). The mean HD95 was 0.77 mm ± 0.31 mm. The distributions of the IoU, DSC, and H95 data were visualized as box plots (Fig. 4B). Like the preoperative CBCT scans, the total volume of the mandible postoperative DL and SA segmentations did not differ significantly (*p* = 0.14). The mean total volume of the mandible segmentations was 21.46 cm^3^ ± 13.68 cm^3^ for the DL segmentations and 21.60 cm^3^ ± 13.75 cm^3^ for the SA segmentations. The mean differences between the volumes of SA and DL segmentations were visualized in a Bland–Altman plot (Fig. 5B). The data are summarized in Table 2.

**Table 2:** Postoperative CBCT segmentation

	DSC^a^	IoU^b^	H95^c^ (mm)	DL volume^d^ (cm^3^)	SA volume^e^ (cm^3^)
Mean	0.96	0.92	0.77	21.46	21.60
St. dev.^f^	0.01	0.02	0.31	13.68	13.75
Minimum	0.95	0.90	0.45	7.36	7.61
Maximum	0.97	0.95	1.46	52.54	53.09
Median	0.96	0.93	0.66	19.31	19.23
*p*-value	na	na	na	0.14^g^	

^a^Dice similarity coefficient, ^b^Intersection over union, ^c^95th percentile Hausdorff distance, ^d^Volume of deep learning segmentation, ^e^Volume of semi-automatic segmentation, ^f^Standard deviation, ^g^Student’s paired samples *t*-test

### Augmented hard tissue segmentation

**Fig. 7:**
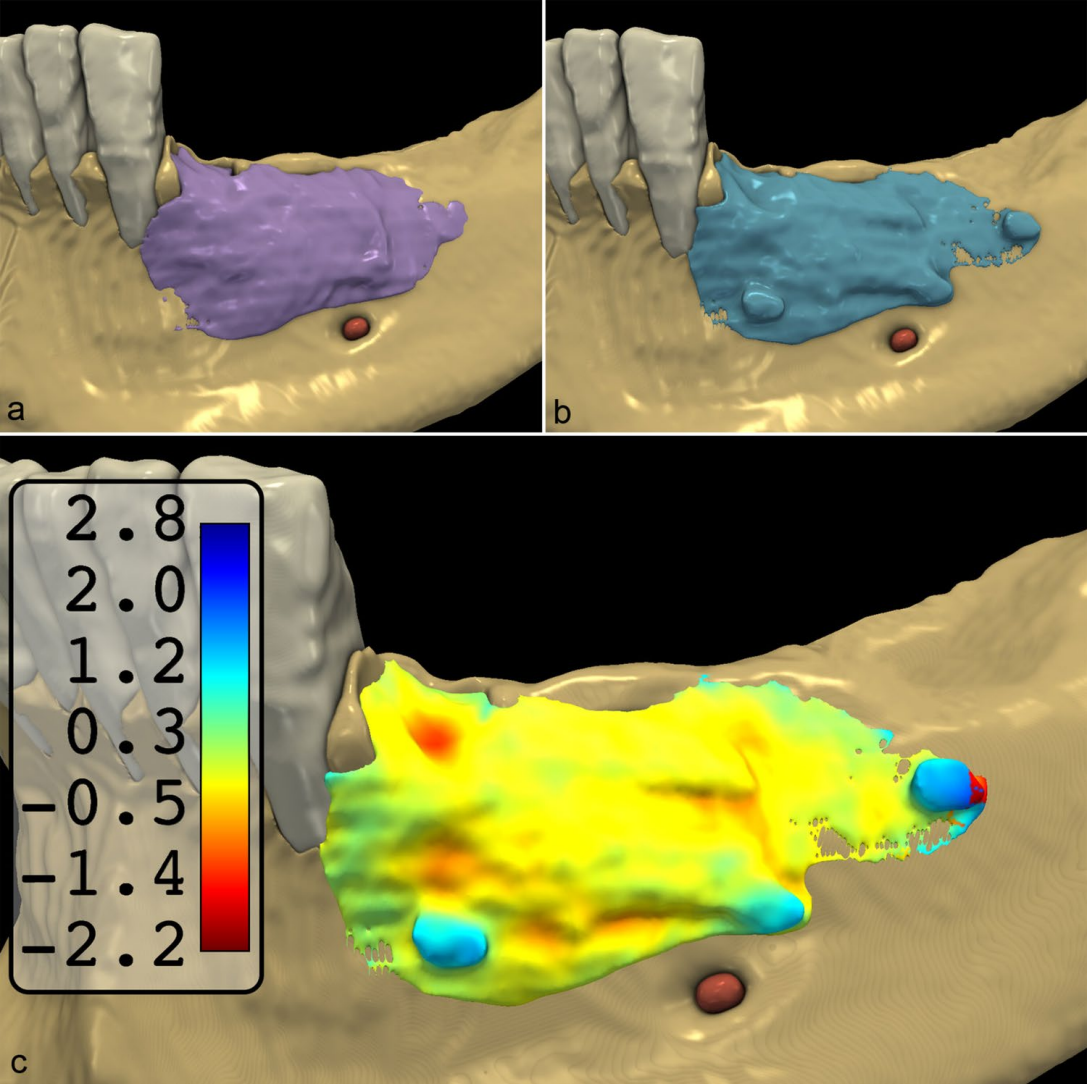
Comparison of augmented hard tissue segmentations obtained by DL and SA segmentations A: SA segmentation results B: DL segmentation results C: Colormap model visualizing the differences between the two methods. Red areas represent negative differences, and blue areas represent positive differences between the reference SA and developed DL models

After spatial alignment, a 3D model of the augmented hard tissues was generated by subtracting preoperative from postoperative segmentation. The volumetric differences of DL and SA segmentations had a mean DSC of 0.85 ± 0.08 and a mean IoU of 0.78 ± 0.07 (Fig. 7). The mean HD95 was 0.91 ± 0.92 mm (Table 3). The distributions of the IoU, DSC, and H95 data were visualized as box plots (Fig. 4C). The mean volume of the augmented hard tissues was 0.45 cm^3^ ± 0.27 cm^3^ for the DL segmentations and 0.48 cm^3^ ± 0.27 cm^3^ for the SA segmentations. Statistical tests indicated that their differences were marginally non-significant (*p* = 0.06). The mean differences between the volumes of SA and DL segmentations were visualized using a Bland–Altman plot (Fig. 5C). The data are summarized in Table 3.

**Table 3:** Augmented hard tissue segmentation

	DSC^b^	IoU^a^	H95^c^ (mm)	DL volume^d^ (cm^3^)	SA volume^e^ (cm^3^)
Mean	0.85	0.78	0.91	0.45	0.48
St. dev.^f^	0.08	0.07	0.92	0.27	0.27
Minimum	0.70	0.70	0.20	0.12	0.12
Maximum	0.96	0.94	3.42	1.03	1.05
Median	0.86	0.76	0.71	0.46	0.51
*p*-value	na	na	na	0.06^g^	

^a^Dice similarity coefficient, ^b^Intersection over union, ^c^95th percentile Hausdorff distance, ^d^Volume of deep learning segmentation, ^e^Volume of semi-automatic segmentation, ^f^Standard deviation, ^g^Student’s paired samples *t*-test

The mean DSC (*p* = 0.34), IoU (*p* = 0.08), and HD95 (*p* = 0.17) did not differ significantly between the preoperative and postoperative segmentations. The data are summarized in Table 4. Out of ten augmentation sites seven was located in the right posterior mandible and three was located in the left posterior mandible.

**Table 4:** Comparison of DL segmentation of pre- and post-operative CBCT scans

	DSC^a^ preoperative	DSC^a^ postoperative	IoU^b^ preoperative	IoU^b^ postoperative	H95^c^ preoperative (mm)	H95^c^ postoperative (mm)
Mean	0.96	0.96	0.92	0.92	0.62	0.77
St. dev.^d^	0.01	0.01	0.02	0.02	0.16	0.31
Minimum	0.93	0.95	0.86	0.90	0.45	0.45
Maximum	0.97	0.97	0.94	0.95	0.91	1.46
Median	0.96	0.96	0.92	0.93	0.56	0.66
*p*-value	0.34^e^		0.08^e^		0.17^e^	

^a^Dice similarity coefficient, ^b^Intersection over union, ^c^95th percentile Hausdorff distance, ^d^Standard deviation, ^e^Student’s paired samples *t*-test

### Timing

The duration of SA mandible segmentation for preoperative CBCT scans averaged 2464.4 s ± 437.98 s (approximately 41 min and 7 s), while postoperative CBCT segmentation took slightly longer, averaging 2554.7 s ± 439.71 s (about 42 min and 35 s). In contrast, DL mandible segmentation was significantly faster, averaging 52.2 s ± 10.82 s for preoperative scans and 53.9 s ± 10.97 s for postoperative scans. This made DL segmentation approximately 47–48 times faster than SA segmentation, with the difference being statistically significant (*p* < 0.0001) for both preoperative and postoperative segmentations.

## Discussion

This study aimed to evaluate the performance of a multi-staged DL model based on the SegResNet architecture [20] to segment CBCT scans acquired before and after horizontal GBR. In addition to preoperative CBCT scans, CBCT scans acquired six months after GBR were also segmented. Therefore, this study could evaluate whether radiographic images of augmented hard tissues and artifacts from the membrane fixation pins impacted DL segmentation accuracy. Our findings are consistent with our previous study [20] and other studies reporting similar DL segmentation frameworks [15, 16, 33]. Comparing the DSC between preoperative and postoperative segmentations (*p* = 0.17, Student’s paired samples *t*-test) indicated that the presence of graft particles and titanium fixation pins in the postoperative CBCT scans did not influence the performance of the SegResNet segmentation network. Therefore, the 3D models of the postoperative CBCT scans produced by DL segmentation are sufficient for subsequent dental implant placement planning. While the DSC between the 3D models of augmented hard tissues obtained through DL and SA segmentation was lower (0.85 ± 0.08) than those of the preoperative (0.96 ± 0.01) and postoperative (0.96 ± 0.01) CBCT scans volumes of augmented hard tissues between DL- and SA segmentations was statistically non-significant (*p* = 0.06). The slightly lower accuracy in segmenting the augmented hard tissue is due to the DL model including the titanium membrane fixation pins in the segmented area. While the titanium pins were mostly included in the postoperative segmentations of the mandible, they can be clearly distinguished from the augmented hard tissues in the radiographic images.

Like our method, Tao et al. [28] have described an automatic segmentation method of bone grafting material after maxillary sinus elevation. They used CBCT scans taken before and six months after sinus augmentation to train a CNN based on a 3D V-Net architecture. They achieved a mean DSC of 0.90 ± 0,25 and a mean IoU of 0.83 ± 0.04 in segmenting the grafted area. These values are comparable but slightly higher than those of augmented hard tissues in our study (DSC = 0.85 ± 0.08, IoU = 0.78 ± 0.07). These differences may be attributed to significant variations in the two CNN models and their functionality. Firstly, Tao et al.’s CNN was trained exclusively on maxillary CBCT scans before and after sinus lift, making the network more robust but limited to segmenting sinus-grafted areas. Secondly, the 3D V-Net segmented only the bone grafts rather than the entire maxilla.

The main limitation of our developed CNN was that compared to similar CNNs, it was trained on a relatively smaller training dataset. The fewer processed CBCT scans included in the training dataset may result in reduced reliability of our network. In order to increase its robustness in future applications, the number of CBCT scans included in the training dataset must be increased. Another notable limitation of our study was that while the developed CNN could segment and classify teeth, its performance in these tasks was not evaluated. Since accurate tooth segmentation is a crucial aspect of creating a virtual patient model and surgical planning, the accuracy of the developed DL segmentation model must be evaluated in the future. In this study, spatial alignment and 3D subtraction were conducted using separate software alongside automatic segmentation. However, our team is currently working on integrating and automating the alignment functionality of “3DSlicer Elastix” [32] and subtraction functionality of “3D Slicer Segment Editor” [7] into our custom application. This integration aims to simplify the process and eliminate the inconvenience of relying on multiple software tools for evaluation. A common challenge with methods involving spatial alignment, segmentation, and subtraction is the potential inaccuracies of the aforementioned steps can compromise evaluation accuracy. Nonetheless, careful inspection of results and occasional manual error corrections can effectively mitigate this limitation. Finally, while the code used in this study is not publicly available due to intellectual property restrictions, this limitation is logistical rather than scientific and does not affect the methodological rigor or the validity of the findings.

Our study only assessed the performance of a DL-based CBCT segmentation. While this is a significant component of acquiring a virtual patient model, there are additional steps in the process that could also be automated. Besides CBCT segmentation, another labor-intensive task in the sequence of virtual patient creation is separating teeth from soft tissues on the intraoral scans to acquire a 3D digital model of the alveolar mucosa/gingiva [34]. Previous studies have successfully utilized point cloud CNN networks to automatically segment intraoral scans [35–37]. Similar to how training datasets for automatic CBCT segmentation are constructed, the training datasets included intraoral scans from fully dentate patients to ensure the robustness of the models. A similar approach could be implemented for automatically segmenting intraoral scans from partially edentulous patients, enabling virtual surgical treatment planning and digital 3D evaluation of GBR procedures at both the hard and soft tissue levels.

## Conclusions

Within the limitations of our study, it can be concluded that the developed multi-stage SegResNet-based DL model produced accurate 3D models of the mandible on CBCT scans taken before and after horizontal GBR. The mean DSC was 0.96 ± 0.01 for preoperative and postoperative CBCT scans, while the volumes did not differ significantly between the DL and SA segmentations. While no significant difference was detected, the segmentation of the augmented hard tissues showed a relatively lower accuracy. This limitation could be overcome by increasing the size of the training dataset. The developed CNN must be retrained in the future to further enhance the robustness and reliability of the segmentation network.

## Data Availability

Data available on reasonable request from the authors.
